# A Rare Case of Schwann Cell Hamartoma in the Duodenum

**DOI:** 10.14309/crj.0000000000000894

**Published:** 2022-11-16

**Authors:** Kasopefoluwa Y. Oguntuyo, Lauren L. Donnangelo, Guangjing Zhu, Stephen Ward, Abhik Bhattacharaya

**Affiliations:** 1 Icahn School of Medicine at Mount Sinai, New York, NY; 2 Division of Gastroenterology, Icahn School of Medicine at Mount Sinai, New York, NY; 3 Department of Pathology, Icahn School of Medicine at Mount Sinai, New York, NY

## Abstract

Mucosal Schwann cell hamartomas (MSCHs) are benign neural lesions that are not associated with inherited syndromes and are primarily found in the distal colon. We report the first case of an MSCH in the duodenum. This case highlights the expansive nature of MSCHs and discusses the implications of this finding in the duodenum and in the context of a hematologic malignancy.

## INTRODUCTION

In their 2009 landmark study, Gibson and Hornick described primarily distal colon lesions from 26 patients, many of whom were undergoing screening colonoscopies. These lesions were solitary with a sessile morphology and 1–6 mm in size with histologic features of spindle cells, wavy nuclei, eosinophilic cytoplasm, and no mitotic activity. Further characterization with immunohistochemistry was notable for S-100 protein positivity without reactivity to markers for other nonadenomatous polyps such as gastrointestinal stromal tumors (KIT), leiomyomas (smooth muscle actin), neurofibromas (CD34), and perineuromas (epithelial membrane antigen or claudin-1). Given this distinct morphological and histological phenotype, Gibson and Hornick proposed the name mucosal Schwann cell hamartomas (MSCHs). This nomenclature also served the additional purpose of distinguishing these benign lesions from malignant lesions and other gastrointestinal neural lesions associated with inherited syndromes such as NF1, MEN2B, or Cowden syndrome.^1^ Since this original case series, many groups have reported cases with colonic MSCH lesions and also in the gastroesophageal junction (GEJ), gastric antrum, gallbladder, and cecum.^2–11^ We present the first MSCH lesion in the duodenum that was incidentally found in a patient with leukemia.

## CASE REPORT

**Figure 1.**
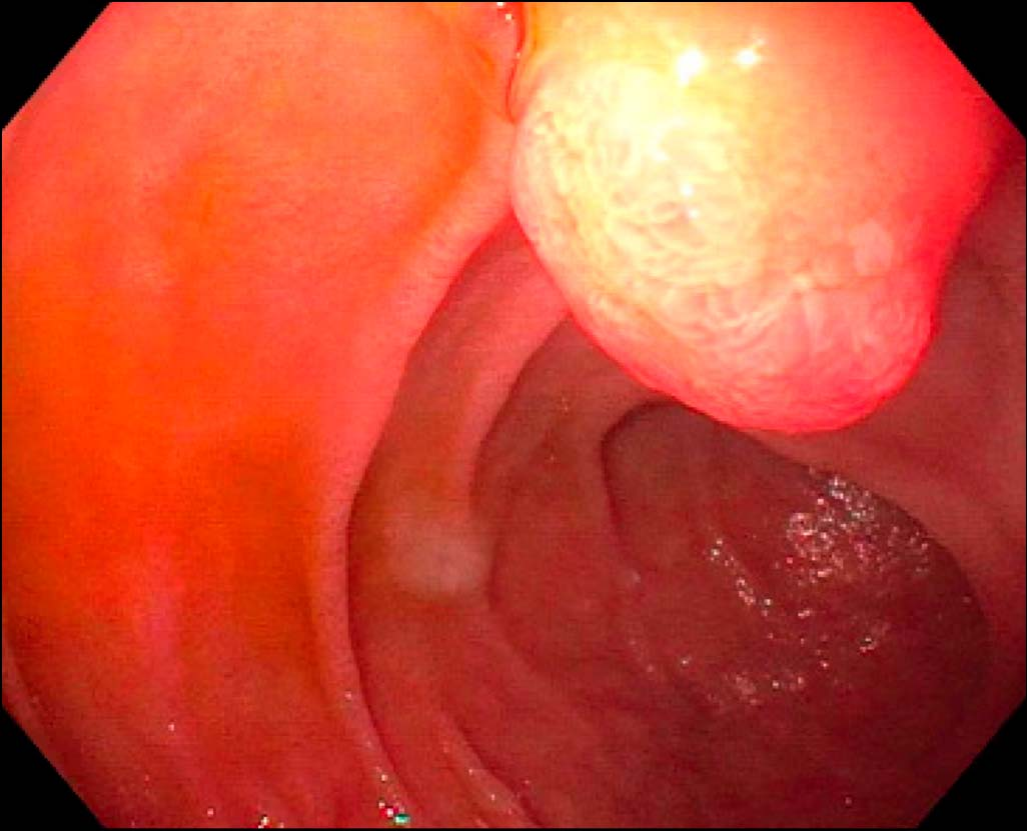
Endoscopic imaging of a 15 mm pedunculated polyp in the first portion of the duodenum.

A 37-year-old woman with a new diagnosis of acute myeloid leukemia with central nervous system involvement and status post leukopharesis, 7 days of cytarabine with short infusions of idarubicin for the first 3 days, and intrathecal chemotherapy and the course complicated by stroke, seizures, and COVID-related respiratory failure status post tracheostomy on a ventilator presented for percutaneous endoscopic gastrostomy tube placement. Upper endoscopy was notable for a 15 mm pedunculated polyp in the first portion of the duodenum (Figure 1). The polyp was removed completely by hot snare polypectomy.

**Figure 2.**
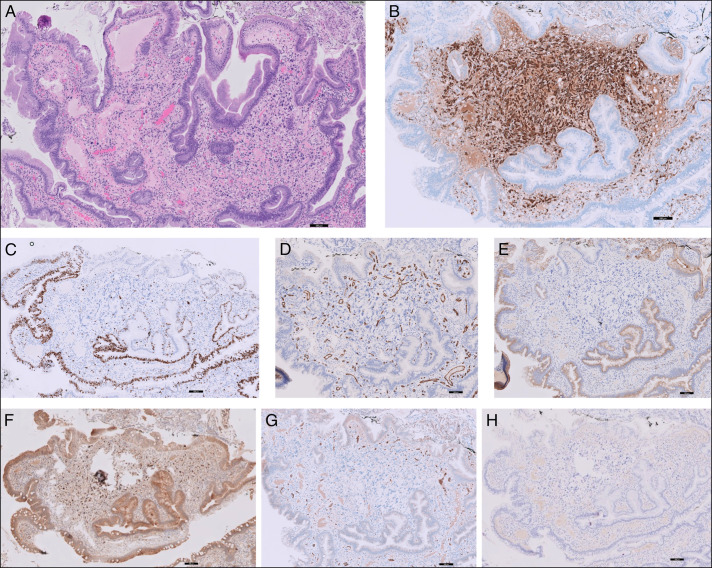
Histological findings from duodenal lesion is consistent with mucosal Schwann cell hamartoma. Hematoxylin and eosin stain with low power magnification (A). Immunohistochemistry for S-100 (B), MIB1/Ki67 (C), CD34 (D), epithelial membrane antigen (E), SOX10 (F), Glut1 (G), and HMB45 (H).

Immunohistochemical staining of the lesion was positive for S-100 and SOX10 and negative for MIB1/Ki67, CD34, epithelial membrane antigen, Glut1, and HMB45 (Figure 2). This profile led to the pathologic diagnosis of duodenal mucosa with Schwann cell hamartoma with degenerative changes. Interestingly, this is the first MSCH case report to show positive staining of SOX10, consistent with its role as a transcription factor in Schwann cells.

Of note, the patient did not have physical examination findings of axillary freckling, inguinal freckling, or café au lait spots that would be suggestive of NF1. In addition, there was no family history of colorectal cancer.

## DISCUSSION

Our incidental finding of an MSCH in the duodenum of a patient undergoing PEG placement for the management of their leukemia extends the gastrointestinal regions susceptible to developing MSCHs. Although the cause of MSCHs has not been determined, it is hypothesized that these lesions may be the result of a reactive process in areas prone to mucosal injury.^6^ This hypothesis is supported by the predominance of MSCHs at the rectosigmoid region and MSCHs at the GEJ^6^ and a retrospective study of 500 cholecystectomies that identified MSCHs in 4% of cases with histological evidence of regional hypertrophy and chronic inflammation.^1–6,10^ Notably, MSCHs found within the GEJ and the gallbladder often lacked the typical polyploid structure found elsewhere in the gastrointestinal tract like the colon or duodenum.^6,10^ This phenotypic difference may suggest either a distinct morphology or perhaps a spectrum of morphologies for MSCHs in different gastrointestinal contexts. Specifically for the duodenum, this polyploid MSCH may be the result of constant exposure to pancreatic digestive enzymes and bile acids.

Moreover, it is uncertain what role the patient's leukemia may have played in the development of the duodenal MSCH. Interestingly, there is one case report of a patient with lymphoma who was found to have a 3 mm MSCH in the transverse colon.^5^ Given the immune system's role in regulating uncontrolled proliferation of cells, it is feasible that the loss of this regulation in both patients allowed for the development of MSCHs. In addition, one case report of a patient with ulcerative colitis identified a 3 mm MSCH in the sigmoid colon, further suggesting that dysregulation of the immune system may play a role in the development of MSCHs.^4^

MSCHs are largely asymptomatic and benign, but there are cases of patients presenting with gastrointestinal/rectal bleeding, loose stools, abdominal pain with intermittent tenesmus, and epigastric pain.^1,7,12,13^ Most of these cases received polypectomies, but one patient had a subtotal gastrectomy with lymph node dissection due to features originally concerning for a gastrointestinal stromal tumor.^7^ As a result, there is a need to consider MSCHs as a diagnosis for polyploid and nonpolypoid lesions throughout the gastrointestinal tract. For the latter, Okamoto et al used narrow band imaging and endoscopic ultrasound to characterize MSCHs that presented atypically as skip-like lesions in the sigmoid colon. With narrow band imaging, granular white opacities were highlighted on the mucosal surface and endoscopic ultrasound highlighted that the lesion was present only in the superficial mucosa.^14^ Although there have been extensive case series and reports characterizing MSCHs, it is still unknown what the true prevalence of these lesions are, what risk factors contribute to the development of these lesions, what mutations drive the proliferation of these cells, and what are the long-term consequences of these lesions.

## DISCLOSURES

Author contributions: KY Oguntuyo wrote the manuscript and is the author guarantor. LL Donnangelo edited the manuscript. G. Zhu provided pathology slides and interpretation of the findings. S. Ward supervised and reviewed the pathological findings. A. Bhattacharaya edited the manuscript and revised the manuscript for intellectual content.

Financial disclosure: None to report.

Informed consent could not be obtained from the family of the deceased. All identifying information has been removed from this case report to protect patient privacy.

## References

[1] Gibson JA Hornick JL. Mucosal Schwann cell “hamartoma”: Clinicopathologic study of 26 neural colorectal polyps distinct from neurofibromas and mucosal neuromas. Am J Surg Pathol. 2009;33(5):781–7. 19065103 10.1097/PAS.0b013e31818dd6ca

[2] Pasquini P Baiocchini A Falasca L . Mucosal Schwann cell “hamartoma”: A new entity? World J Gastroenterol. 2009;15(18):2287–9. 19437573 10.3748/wjg.15.2287 PMC2682248

[3] Rocco EG Iannuzzi F Dell'Era A . Schwann cell hamartoma: Case report. BMC Gastroenterol. 2011;11:68. 21663626 10.1186/1471-230X-11-68 PMC3123296

[4] Neis B Hart P Chandran V Kane S. Mucosal Schwann cell hamartoma of the colon in a patient with ulcerative colitis. Gastroenterol Hepatol (N Y). 2013;9(3):183–5. 23961270 PMC3745209

[5] Bae MN Lee JE Bae SM . Mucosal schwann-cell hamartoma diagnosed by using an endoscopic snare polypectomy. Ann Coloproctol. 2013;29(3):130–4. 23862132 10.3393/ac.2013.29.3.130 PMC3710775

[6] Li Y Beizai P Russell JW Westbrook L Nowain A Wang HL. Mucosal Schwann cell hamartoma of the gastroesophageal junction: A series of 6 cases and comparison with colorectal counterpart. Ann Diagn Pathol. 2020;47:151531. 32460039 10.1016/j.anndiagpath.2020.151531

[7] Hytiroglou P Petrakis G Tsimoyiannis EC. Mucosal Schwann cell hamartoma can occur in the stomach and must be distinguished from other spindle cell lesions. Pathol Int. 2016;66(4):242–3. 26778643 10.1111/pin.12376

[8] Sharma K Dhua AK Goel P Jain V Yadav DK Ramteke P. Mucosal Schwann cell hamartoma of the gall bladder. J Indian Assoc Pediatr Surg. 2021;26(3):182–3. 34321790 10.4103/jiaps.JIAPS_45_20 PMC8286015

[9] Ismael F Khawar S Hamza A. Mucosal Schwann cell hamartoma of the gallbladder. Autops Case Rep. 2021;11:e2021338. 34722355 10.4322/acr.2021.338 PMC8552972

[10] Khanna G Ghosh S Barwad A Yadav R Das P. Mucosal Schwann cell hamartoma of gall bladder: A novel observation. Pathology. 2018;50(4):480–2. 29739615 10.1016/j.pathol.2017.11.095

[11] Chintanaboina J Clarke K. Case of colonic mucosal Schwann cell hamartoma and review of literature on unusual colonic polyps. BMJ Case Rep. 2018;2018:bcr2018224931. 10.1136/bcr-2018-224931 PMC6157549 30244220

[12] Bae JM Lee JY Cho J Lim SA Kang GH. Synchronous mucosal Schwann-cell hamartomas in a young adult suggestive of mucosal Schwann-cell harmatomatosis: A case report. BMC Gastroenterol. 2015;15:128. 26444007 10.1186/s12876-015-0349-4 PMC4596299

[13] Klair JS Girotra M Agarwal A Aduli F. Mucosal Schwann cell hamartoma: Just benign or more? Int J Colorectal Dis. 2014;29(12):1597–8. 25015570 10.1007/s00384-014-1954-3

[14] Okamoto T Yoshimoto T Fukuda K. Multiple non-polypoid mucosal Schwann cell hamartomas presenting as edematous and submucosal tumor-like lesions: A case report. BMC Gastroenterol. 2021;21(1):29. 33468041 10.1186/s12876-021-01607-w PMC7816477

